# Acupuncture as Adjuvant Therapy for Treating Stable Angina Pectoris with Moderate Coronary Artery Lesions and the Mechanism of Heart-Brain Interactions: A Randomized Controlled Trial Protocol

**DOI:** 10.1155/2021/6634404

**Published:** 2021-04-28

**Authors:** Long Zhao, Qingqiao Song, Huaqin Wu, Yanli Wang, Jiani Wu, Jiliang Fang, Zhigang Li

**Affiliations:** ^1^School of Acupuncture, Moxibustion and Tuina, Beijing University of Chinese Medicine, Beijing 100029, China; ^2^Guang'anmen Hospital, China Academy of Chinese Medical Sciences, Beijing 100053, China

## Abstract

*Background*. Stable angina pectoris with moderate coronary artery lesions is a syndrome caused by coronary artery stenosis, which endangers the quality of life. Previous acupuncture studies have shown effectiveness as a complementary therapy for ischaemic heart disease. However, more clinical evidence is needed for verification, and the mechanism should be investigated, especially involving the functional interactions between the heart and brain. Therefore, we designed a clinical trial to provide more evidence for acupuncture efficacy and its mechanism in ischaemic heart disease. *Methods/Design*. A total of 80 participants will be randomized to the electroacupuncture group and sham-electroacupuncture group at a ratio of 1 : 1. This trial will be conducted over 8 weeks, including a 2-week screening, 2-week treatment, and 4-week follow-up. All participants will continue to receive similar basic disease treatment procedures before the trial (including lifestyle changes and treatment for standard supportive medications, hypertension, and hyperlipidaemia, such as aspirin, metoprolol succinate, atorvastatin, and sodium fosinopril). Additionally, 12 sessions of acupuncture will be administered during the treatment period. The main outcome is Seattle Angina Questionnaire scores. The other observation indices are the heart rate variability and self-rating anxiety scale and self-rating depression scale scores. To explore mechanisms based on the hypothesis of a correlation between heart and brain function, fMRI scans will be used to detect functional brain changes in 15 patients from each group at baseline and at the end of treatment. Finally, the efficacy of acupuncture will be evaluated, and the HRV and imaging data will be correlated with clinical data to investigate the possible relationships between the brain and heart activity. *Discussion*. This trial will provide evidence for acupuncture as adjuvant therapy for the treatment of stable angina pectoris with moderate coronary artery lesions. The results will shed light on potential mechanisms of heart-brain interactions underlying acupuncture as an adjuvant therapy for treating ischaemic heart disease. Trials registration: Clinical Trial, https://clinicaltrials.gov/ct2/show/ChiCTR1900024937. Registered 4 August 2019, http://www.chictr.org.cn/.

## 1. Introduction

As a worldwide public health problem, coronary artery atherosclerosis is quite common in clinical practice. Moderate coronary artery lesions are defined as coronary stenosis between 40% and 70% by coronary artery angiography [1]. Although these lesions are not usually considered as serious enough to warrant percutaneous coronary interventions (PCI), they are associated with plaque rupture or erosion and an increased risk of acute cardiac events [2] and are an independent risk factor for cardiogenic death [3]. Stable angina pectoris is a common cardiovascular symptom that coexists with coronary stenosis and is characterized by chest pain or discomfort in the left chest or adjacent areas caused by myocardial ischaemia [4]. Acupuncture treatment has been reported to alleviate the incidence of stable angina pectoris.

Acupuncture, as a widely used complementary and alternative therapy, originated in ancient China [5]. At present, acupuncture has been recognized as an alternative treatment for various diseases in 183 countries [6]. Manual and electrical acupuncture are two currently used kinds of acupuncture. Compared to traditional manual acupuncture, electroacupuncture employs a small electric current to stimulate the metal needle in the acupoint of the body [7]. Experience has accumulated for acupuncture in treating stable angina pectoris in recent years. Evidence has emerged that it can alleviate symptoms, reduce anginal attacks, and decrease nitroglycerin use [8–11]. Animal studies have shown that acupuncture can protect the ischaemic myocardium and improve cardiac function [12–14]. Furthermore, experimental research has also revealed that acupuncture alters electrical and mechanical cardiac activities, strengthens myocardial substrate metabolism in the ischaemic region, and minimizes myocardial ischaemic injury [15]. However, these preliminary results need more evidence to establish the efficacy and mechanism [16].

Over the past years, brain-heart interactions have been shown to have important potential implications for the treatment of cardiovascular disease. For example, cerebrovascular accidents and transient brain ischaemic attacks are often caused by arrhythmias or congestive heart failure [17–19]. Conversely, cerebrovascular dysfunction may cause heart rhythm disorders detectable by electrocardiography (ECG) [20,21]. Furthermore, patients with heart disease have a high incidence of neurological or psychiatric symptoms, including depression and/or depressive symptoms and memory disorders [22]. As a result, a new interdisciplinary field integrating the mind and heart recently emerged.

It is well known that the heart receives signals from the brain through the sympathetic and parasympathetic nerves of the central autonomic network [23,24]. The sympathetic nervous system mainly regulates many physiological processes, including regulation of temperature, blood pressure, respiration, and heart rate. The vagus nerve is the primary composite of parasympathetic nerves, which innervate the thoracic and abdominal organs involving the cardiovascular, respiratory, gastrointestinal, and endocrine systems [25]. Heart rate variability (HRV) is an index of the balance between the sympathetic and parasympathetic nervous systems, and it is used to monitor cardiac vagal function [26,27].

In previous studies, clinical and experimental evidence suggested that angina pectoris may be mediated by coronary chemoreceptors and transmitted to the brain through vagal afferent nerves [28]. The central nervous system may serve as an essential node of acupuncture treatment for moderate coronary artery lesions with stable angina pectoris; however, this possibility has rarely been investigated. The direct interrelationships of the nervous system, the brain, and the heart have increasingly become an important focus of research [29]. When these relationships are better clarified, more appropriate treatments can be introduced, which would be beneficial for patients with cardiovascular and cerebrovascular comorbidities [30].

In recent years, functional magnetic resonance imaging (fMRI) has been widely used in brain and clinical neuroscience research. It has become a hot research topic for exploring the mechanisms of acupuncture [31–33]. Stable angina pectoris was reported to be closely related to brain dysfunction [34]. Whether the effects of acupuncture on stable angina pectoris with moderate coronary artery lesions are correlated with modulation of cerebral activity remains unclear and is worth further investigation.

Therefore, we designed a randomized controlled trial to test the therapeutic effects of acupuncture on stable angina pectoris with moderate coronary artery lesions and to explore the potential mechanisms of heart-brain interactions by combining HRV and fMRI assessments.

## 2. Methods/Design

### 2.1. Design

**Figure 1:**
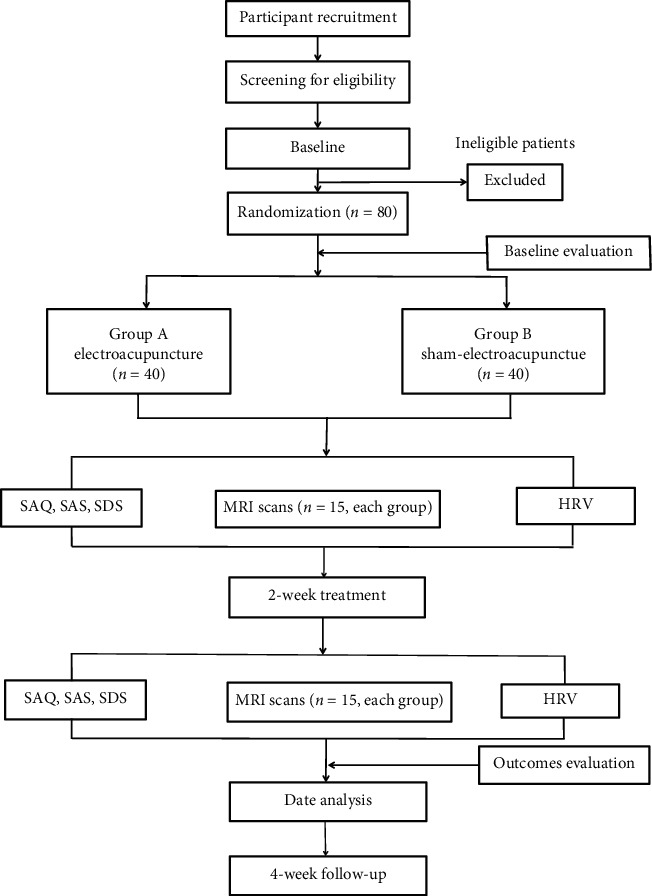
Flow chart.

The study is a randomized controlled clinical trial, and a total of 80 patients who are diagnosed with stable angina pectoris with moderate coronary artery lesions will be recruited and randomly assigned to two treatment groups (electroacupuncture or sham-electroacupuncture group) in a 1 : 1 ratio. Fifteen patients in each group will be selected to undergo MRI scanning. Outcome assessments will be performed at two timepoints: baseline and the end of treatment (Figure 1, Table 1). Eligible patients will be enrolled from Guang An Men Hospital, China Academy of Chinese Medical Sciences in Beijing, China.

### 2.2. Randomization and Blinding

In the study, we will use the method of complete randomization. The randomization numbers will be generated by the statistical analysis software. Randomization numbers will be managed by statisticians who are not involved in the study.

The investigators and patients will be blinded to group allocation. Eighty patients will be randomly divided into two groups according to a simple computer-generated randomization number. To ensure that the patients remain blinded, both groups will be connected to an electroacupuncture apparatus after the needle is applied. The difference is that the apparatus in the electroacupuncture group is set with the current on and the sham-electroacupuncture group is set with the current off. Fifteen patients will be randomly selected from each group to undergo fMRI scanning. During the acupuncture treatment, patients in each group receive treatment alone to refrain from communication.

### 2.3. Blinding Assessment

Blinding assessment will be evaluated after the end of the study. To evaluate the success of blinding, a blinding index will be assessed after the final treatment. Blinding will be maintained until the study is finished.

## 3. Patients

### 3.1. Study Population

We will select stable angina patients with moderate coronary artery lesions (40%–70%). Patients who voluntarily provide informed consent and meet the inclusion/exclusion criteria will be included in the study. The inclusion criteria and exclusion criteria include the following.

### 3.2. Inclusion Criteria

(1) Coronary angiography results show coronary artery stenosis of 40% to 70%. (2) Patients meet the diagnostic criteria of the American College of Cardiology/American Heart Association (ACC/AHA), with a duration of more than 3 months and a frequency of angina attack of more than twice a week. (3) Patients are 30–80 years of age and male or female. (4) Blood pressure and glucose levels of the patient are stable. (5) Patients are right-handed. (6) Patients have not participated in any other clinical trial in the past three months. (7) Patients agree to provide written informed consent.

### 3.3. Exclusion Criteria

Patients matching one of the following conditions will be excluded. (1) The results of coronary angiography suggest that coronary artery stenosis is <50% or >70%. (2) Patient's age is ≤ 30 or ≥80. (3) Patients have complex cardiovascular, digestive, respiratory, urinary, blood, nervous system, or endocrine system disease or other serious primary diseases, and the treatment is not effective. (4) Patients have acute coronary syndromes, severe arrhythmias, tachycardia, and premature beats, atrial and ventricular fibrillation, primary cardiomyopathy, or valvular heart disease. (5) Blood pressure and blood glucose are not being appropriately managed. (6) Women are pregnant or lactating. (7) Patients have severe depressive or anxiety disorder. (8) Patients received acupuncture treatment for cardiovascular disease in the past three months. (9) Patients are participating in other clinical trials. (10) Patients have contraindications to MRI scans such as claustrophobia, tattoo, or implanted ferromagnetic metal.

### 3.4. Recruitment Procedures

The following methods will be used to recruit patients. The investigators will recruit eligible participants from the outpatient and inpatient populations using advertisements. Cardiologists will recommend that they participate in the trial. The investigator screens qualified participants based on inclusion/exclusion criteria and continues the recruitment process.

### 3.5. Interventions

To ensure the safety of participants and improve the prognosis of patients, we follow the European and Chinese guidelines for the management of patients who have stable angina pectoris [35]. All participants in the two groups will receive the same basic treatment with short-term sublingual nitroglycerin therapy when the patient suffers acute angina pectoris symptoms.

### 3.6. Basic Management

Basic management includes health education and standard medications during the trial. Health education includes suggestions for lifestyle changes, such as increased physical activity, decreased alcohol consumption, weight reduction, and smoking cessation. Standard supportive medications include aspirin, *β*-blockers, statins, and angiotensin converting enzyme inhibitors [36]. The drugs used in the study will be prepared as follows: aspirin, 100 mg QN; metoprolol succinate, 47.5 mg QD; atorvastatin, 20 mg QN; and fosinopril sodium, 10 mg QD.

### 3.7. Acupoint Selection

According to traditional acupuncture theory and the literature, acupoints used for angina pectoris are mainly on cardiac meridians and pericardial meridians. The following acupoints will be used in this study: Ximen (PC4) and Neiguan (PC6). The name/code and location of all acupuncture points are in compliance with World Health Organization (WHO) standards [37]. Ximen (PC4) is on the palmar aspect of the forearm, 5.0 cun above the transverse crease of the wrist and between the tendons of the palmaris longus and flexor carpi radialis muscles; Neiguan (PC6) is on the palmar aspect of the forearm, 2.0 cun above the transverse crease of the wrist and between the tendons of palmaris longus and flexor carpi radialis muscles. All acupuncture points are used bilaterally.

### 3.8. Experimental Protocol

The acupuncture protocol is derived from the clinical practice of Chinese medicine and revised based on the opinions of experienced acupuncturists. The patients in each group will receive a total of 12 sessions of acupuncture over 2 weeks with 6 sessions per week (once a day, followed by a one-day interval). Each group will receive the same basic treatment, including health education and essential medications. The entire study period is 8 weeks, which includes a 2-week baseline, 2-week treatment, and 4-week follow-up period. The acupuncturists who will deliver the treatment procedures are qualified TCM practitioners with at least 2 years of acupuncture experience. All acupoints were stimulated by single-use disposable stainless steel needles (0.35 mm × 40 mm; Beijing Zhongyan Taihe Medical Instrument Co., Ltd., Beijing, China). Needles will be perpendicularly inserted into acupoints at a depth of 20–30 mm after sterilization of the skin with 75% alcohol. Then, the acupuncturist will manipulate the needles by lifting and inserting techniques with an amplitude of 3–5 mm combined with twirling and rotation at 90°–180° until the patient feels numbness, soreness, distention, or heaviness (viz., the “deqi” sensation). Then, the needle holders will be connected to paired electrodes from the electroacupuncture apparatus (HWATO-SDZ-IIB, Suzhou Medical Appliance Factory, Suzhou city, China). The electroacupuncture group will receive further stimulation of the acupoint for 30 minutes with 2 Hz sparse wave-induced stimulation. The electrical stimulation intensity will be adjusted from 0.1 mA to 2.0 mA to avoid pain sensations. For the sham-electroacupuncture group, the electroacupuncture apparatus will be set without the current on, and the needle will be retained in place for 30 minutes. In the case of an angina pectoris attack emergency, participants will be instructed to take one kind of medication based on past treatment history and personal contraindications. Basically, we recommend that all patients use nitroglycerin. Regardless of the kind of medication, participants will be required to record the medication details, including the name of the medication, dose, frequency, and administration time. Other antianginal medications are prohibited, and if taken, the participants will be excluded from the analysis for violating the agreement.

### 3.9. Outcome Measurement

All outcomes will be measured at baseline and at the end of treatment. The primary outcome will be Seattle Angina Questionnaire (SAQ) scores, and the secondary outcomes will include HRV, the self-rating anxiety scale (SAS), and the self-rating depression scale (SDS).

At baseline, participants will undergo a physical examination and lab tests, including blood and urine tests—blood pressure, blood glucose, and blood lipid tests—and liver and kidney function tests.

### 3.10. MRI Data Acquisition for the Functional Brain Imaging Study

At baseline and at the end of the treatment period, participants will undergo an MRI scan with a 3.0 T MR scanner (Skyra, Siemens Medical Company) at Guang An Men Hospital, China Academy of Chinese Medical Sciences, Beijing, China. The scanning procedure contains a localizer, high-resolution three-dimensional T1-weighted imaging (3D–T1W1), and a resting-state scan with blood oxygenation level-dependent fMRI (BOLD–fMRI). The 3D–T1W1 anatomical scanning parameters will be as follows: echo time (TE) = 2.98 s, repetition time (TR) = 2530 ms, field of view (FOV) = 256 mm × 256 mm, flip angle = 7°, slice thickness = 1.0 mm, and voxel size = 1.0 mm × 1.0 mm × 1.0 mm. The BOLD–fMRI scanning parameters will be as follows: TE = 30 s, TR = 2000 ms, FOV = 224 mm × 224 mm, flip angle = 90°, slice thickness = 3.5 mm with no gap, and voxel size = 3.5 mm × 3.5 mm × 3.5 mm.

Brain function data will be acquired from the patient in a state of nonacupuncture with eyes closed, no specific thinking activity, and sound-proof earplugs to block noise. A standard 20-channel head coil with a suppression foam pad will be used to fix head motion. Patients in the electroacupuncture group will receive the scans in the following order: resting-state BOLD–fMRI, 3D–T1WI, and second BOLD–fMRI after electroacupuncture treatment for 30 minutes. Patients in the sham-electroacupuncture group will receive scans in the same order with the same parameters but without current stimulation.

### 3.11. Safety and Adverse Events

The risk of side effects of acupuncture treatment is very low based on clinical practice and the literature. All adverse events related to acupuncture will be recorded during the treatment and follow-up periods. These rare adverse events include bleeding, haematoma, fainting, severe pain, and local infections. In addition, adverse events related to the basic therapeutic medications being administered will also be recorded. Serious adverse events (SAEs) are defined as death or life-threatening events that may require hospitalization or lead to persistent or severe disability/incapacity. If a participant suffers from any adverse/serious adverse events, the relevant treatments will be administered, and all details will be recorded and reported. In addition, SAEs will be reported immediately to the ethics committee so that they can decide whether the patient should withdraw from the trial.

### 3.12. Quality Control

To improve the reliability and repeatability of the research results, quality control will be carried out every 2 months and inspected by specifically trained medical staff throughout the study. In this trial, quality control will be strengthened not only from clinical treatment but also from fMRI data acquisition. Acupuncture treatment will be performed by the same acupuncturist. To avoid interference from unstable physiological and psychological factors, patients will be asked to maintain their regular lifestyle and avoid staying up late and ingesting alcohol for 24 hours before scans. Before scanning, every patient will be evaluated by the SDS and SAS. Moreover, the scanning process will be conducted in the same scanner.

### 3.13. Sample Size Calculation and Statistical Analysis

#### 3.13.1. Sample Size

According to a previous systematic review [38], the average frequency of angina pectoris attacks is approximately 10.6 weekly events before acupuncture treatment. The average frequency of angina pectoris attacks is approximately 6.1 weekly events after acupuncture treatment. The difference based on acupuncture efficacy is 4.5. Considering a standard deviation of 5.5, *α* = 0.05, and 1-*β* = 0.9, the sample size was calculated based on the following formula.

(1) $$\begin{array}{c} N1=N2=\frac{{\left(Z_{1-\alpha }+Z_{1-\beta }\right)}^{2}\delta^{2}\left(1+1/K\right)}{{\left(U_{T}-U_{C}-\Delta \right)}^{2}},\\ Z\left(1-\alpha \right)=1.960,\\ Z\left(1-\beta \right)=1.282. \end{array}$$

Considering a 20% dropout rate during the study, a total of 80 patients will be finally recruited, with 40 patients in each group.

The sample size calculation for neuroimaging studies is different from that for classic randomized controlled trials. Based on previous neuroimaging studies [39–41], 12–15 participants in each group are a reasonable sample size to assess stable cerebral responses. In this study, 15 participants randomly selected from each group will undergo MRI scanning, which is consistent with the current design of neuroimaging studies and results in stable and reliable test efficacy.

#### 3.13.2. Statistical Analysis

In this study, the statistical analysis of the clinical data will be performed by a statistician, who will be blinded to the trial, by using SPSS 19.0 statistics software. Demographic data and some basic indicators will be analysed to measure the balance of the two groups at baseline. Continuous variables will be expressed as the means ± standard deviation (SD). Categorical variables will be expressed as numbers and percentages (%). Categorical variables will be analysed with the chi-square (*X*^2^) test. Continuous variables will be analysed with two-way repeated measures ANOVA and Kruskal–Wallis ANOVA. Statistical analysis between the two groups will be performed using Dunnett's test. The comparison between baseline and end of treatment in each group will be carried out with a paired samples *t*-test. Statistical significance will be set to *P* < 0.05 with the two-sided test.

For the BOLD–fMRI data processing, we will use the Statistical Parametric Mapping software SPM12 (http://www.fil.ion.ucl.ac.uk/spm) with the MATLAB platform of DPABI 4.3 software to process and analyse whole-brain functional areas and the differences in the resting-state default network before and after treatment. The main analytical methods for cerebral responses to the different interventions include regional homogeneity (ReHo), amplitude of low-frequency fluctuations (ALFF), and seed-based functional connectivity based on the results of ReHo and ALFF.

## 4. Discussion

Stable angina pectoris with moderate coronary artery lesions is a common disease that endangers people's health and quality of life [42–44]. Many studies have shown that most cardiovascular events, especially acute myocardial infarction (AMI), may correlate with coronary artery stenosis [45,46]. The harmonious bidirectional interaction between the heart and brain is a necessary prerequisite for retaining normal physiological function and the state of the heart [47]. However, disharmony in the heart-brain relationship may be one of the critical pathogeneses of stable angina pectoris. Acupuncture points are closely related to the activities of the nervous system and especially related to specific cerebral cortical functions [48]. Acupuncture may be beneficial for stable angina pectoris by regulating brain activity [34].

Although previous studies have confirmed the efficacy of acupuncture treatment for stable angina pectoris, most studies have been restricted to animal experiments, and the mechanism of acupuncture has mainly been confined to the local responses near acupoints and peripheral cardiac responses [16]. Some studies have shown that the corresponding brain regions are involved in the regulation of an integrated central nervous system mechanism of acupuncture treatment for angina pectoris, indicating that the cardiocerebral axis and autonomic nervous system are closely related to cardiovascular disease and that acupuncture may adjust the cardiocerebral axis [49]. Brain function imaging has been conducted to evaluate the effectiveness of cardiac autonomic nerve function in healthy participants. However, the sample size was insufficient, and the results were less repeatable [50]. Few studies have investigated the correlation among clinical symptoms and heart and brain function. Further studies are necessary to elucidate the mechanisms connecting the local and central effects of acupuncture.

Our current study will provide evidence of acupuncture as adjuvant therapy for treating ischaemic heart disease and improve our understanding of the mechanisms underlying heart-brain interactions.

## Figures and Tables

**Table 1:** Study schedule.

Period	Baseline	Screening	Treatment	Follow-up
Week	−2	0	2	4
Informed consent	x			
Inclusion/exclusion criteria		x		
Medical history	x			
Concomitant disease/medication	x	x	x	x
Physical examination	x	x	x	
CCS angina class		x	x	
SAQ scoring		x	x	
HRV		x	x	
SAS and SDS scoring		x	x	
MRI scan		x	x	
Blood glucose, pressure, and lipid tests		x		
Blood, liver, and renal function tests		x		
Adverse events			x	
Patient compliance		x	x	x
Reasons of withdrawals			x	x
Safety evaluation		x	x	x

CCS, Canadian Cardiovascular Society; HRV, heart rate variability; SAQ, Seattle Angina Questionnaire; SAS, self-rating anxiety scale; SDS, self-rating depression scale.

## Data Availability

The data used to support the findings of this study have not been made available because the research is currently not finished. We will publish it after the end of the study.

## Abbreviations

ECG: Electrocardiography
fMRI: Functional magnetic resonance imaging
CCS: Canadian cardiovascular society
HRV: Heart rate variability
SAQ: Seattle angina questionnaire
SAS: Self-rating anxiety scale
SDS: Self-rating depression scale
QN: Quaque nocte
QD: Quaque die
SAE: Serious adverse events
SD: Standard deviation
ANOVA: Analysis of variance
ReHo: Regional homogeneity
ALFF: Amplitude of low-frequency fluctuations
AMI: Acute myocardial infarction.
